# Association between preoperative grip strength and postoperative upper extremity impairments in patients with breast cancer: a retrospective cohort study

**DOI:** 10.1007/s12282-025-01699-2

**Published:** 2025-04-10

**Authors:** Mayu Mizuta, Maho Okumura, Junichiro Inoue, Yuya Ueda, Shin Kondo, Mayuko Miki, Tomonari Kunihisa, Rei Ono, Yoshitada Sakai, Toshihiro Akisue

**Affiliations:** 1 Division of Rehabilitation, Kobe University Hospital, 7-5-2, Kusunoki-cho, Chuo-ku, Kobe, Hyogo 650-0017 Japan; 2 Division of Rehabilitation Medicine, Kobe University Hospital International Clinical Cancer Research Center, 1-5-1, Minatojimaminamimachi, Chuo-ku, Kobe, Hyogo 650-0047 Japan; 3 Department of Rehabilitation Science, Kobe University Graduate School of Health Sciences, 7-10-2, Tomogaoka, Suma-ku, Kobe, Hyogo 654-0142 Japan; 4 Division of Rehabilitation, Tokushima University Hospital, 2-50-1, Kuramoto-cho, Tokushima, Tokushima 770-0042 Japan; 5 Division of Breast and Endocrine Surgery, Department of Surgery, Kobe University Hospital and Graduate School of Medicine, 7-5-2, Kusunoki-Cho, Chuo-ku, Kobe, Hyogo 650-0017 Japan; 6 Health and Nutrition Department of Physical Activity Research, National Institutes of Biomedical Innovation, KENTO Innovation Park NK Bldg 3-17, Senriokashinmachi, Settu, Osaka 566-0002 Japan; 7 Division of Rehabilitation Medicine, Kobe University Graduate School of Medicine, 7-5-2, Kusunoki-cho, Chuo-ku, Kobe, Hyogo 650-0017 Japan

**Keywords:** Grip strength, Upper extremity impairments, Breast cancer

## Abstract

**Background:**

Upper extremity impairments in patients with breast cancer persist after curative surgery. Although postoperative factors associated with upper extremity impairments have been reported, modifiable factors affecting these impairments preoperatively remain unclear. This study aimed to investigate the relationship between preoperative grip strength and postoperative upper extremity impairments in patients with breast cancer.

**Methods:**

This retrospective cohort study included patients (age ≥ 18 years) with breast cancer who underwent mastectomy. Maximum grip strength was measured on the day before surgery. Upper extremity impairments were assessed 4–16 months after surgery using the Disabilities of the Arm, Shoulder and Hand (DASH) scale. Multiple linear regression analysis was used to evaluate the association between preoperative grip strength and postoperative upper extremity impairments.

**Results:**

In total, 72 patients were included in the analysis. Multiple linear regression analysis showed that preoperative grip strength was significantly associated with the postoperative DASH score after adjusting for confounding factors (*β* = − 1.27, 95% confidence interval − 2.08 to − 0.48, *p* = 0.002).

**Conclusions:**

This study showed that low preoperative grip strength is a risk factor for postoperative upper extremity impairments in patients with breast cancer. Providing prehabilitation to maintain and improve muscle strength immediately after diagnosis is important. Moreover, an individualized follow-up protocol according to preoperative screenings to prevent postoperative upper extremity impairments is necessary.

**Supplementary Information:**

The online version contains supplementary material available at 10.1007/s12282-025-01699-2.

## Introduction

Breast cancer is the most prevalent cancer in women [1]. The 5-year relative survival rate of patients with breast cancer is approximately 90% owing to advances in treatment [2, 3], where long-term survival after diagnosis is expected. Thus, it is important to improve the long-term quality of life (QOL) of patients with breast cancer after treatment [4, 5]. Although current surgical options for breast cancer are minimally invasive, long-term upper extremity impairments (such as restricted range of motion in the affected shoulder, pain, reduced muscle strength, and lymphedema) persist after resection [6–9]. These impairments can affect the physical, psychological, and social aspects of a patient’s life and result in poor QOL [10–12]. Therefore, it is essential to focus on upper extremity impairments to improve the postoperative QOL of patients with breast cancer.

Age, body mass index (BMI), surgical approach (such as axillary lymph-node dissection), and neoadjuvant or adjuvant treatment (such as chemotherapy and radiation therapy) are risk factors for upper extremity impairments in patients with breast cancer [7, 13]. Furthermore, these patients experience a decline in physical activity, physical function, and mental function shortly after diagnosis [14–16], adversely affecting postoperative recovery and symptoms [17, 18]. Thus, preoperative interventions for physical activity, physical function, and mental function may be critical in the perioperative management of patients with breast cancer. In addition, identifying pre-treatment modifiable variables that may affect post-treatment upper extremity impairments is necessary to prevent or inhibit the worsening of the impairments in these patients immediately after diagnosis.

Grip strength has been suggested as a risk factor for upper extremity impairments; however, a previous study revealed only an association between postoperative grip strength and upper extremity impairments in patients with breast cancer [19–21]. Grip strength is an outcome that can be measured easily in clinical settings [22] and indicates global muscle strength [23–25]. Although muscle strength is believed to be a variable factor that can be improved via intervention [26], no study has investigated the association between preoperative grip strength and postoperative upper extremity impairments in this demographic.

Therefore, this study aimed to investigate the association between preoperative grip strength and postoperative upper extremity impairments in patients with breast cancer.

## Patients and methods

### Study design and participants

This was a retrospective cohort study conducted at a single university hospital. We recruited 149 patients with breast cancer, aged ≥ 18 years, and who underwent mastectomy between October 2021 and September 2022. Patients who declined to provide consent, had bilateral breast cancer, underwent breast reconstruction (besides a primary one-stage reconstruction), certified as requiring nursing care before surgery, had a physical disability certificate, or had missing data were excluded from the study. The study protocol was approved by the Ethics Committee of Kobe University Graduate School of Medicine (B220153-H). The study was conducted in accordance with the Declaration of Helsinki. All participants were given the opportunity to opt out of this study.

### Measurements

#### Grip strength

Grip strength was measured using a Jamar dynamometer (TKK5401 Grip-D; Takei, Niigata, Japan) by a physical therapist on the day before surgery. Patients were instructed to hold the dynamometer as tightly as possible while standing. The maximum value of two measurements on the affected side was used in the analysis.

#### Upper extremity impairments

Upper extremity impairments were assessed using the Disabilities of the Arm, Shoulder and Hand (DASH) scale [27]. The DASH scale consists of 30 questions: 25 regarding disability and 5 on symptoms [28, 29]. The DASH scale scores range from 0 to 100, with a higher score indicating greater upper extremity impairments. DASH measurements were performed 4–16 months postoperatively.

#### Other variables

The following variables were obtained from the patients’ medical records: age, sex, BMI, matching of the dominant and affected sides (yes/no), comorbidities (high blood pressure, diabetes mellitus, and musculoskeletal disease), clinical stage, postoperative period, neoadjuvant chemotherapy (yes/no), neoadjuvant hormonal therapy (yes/no), adjuvant chemotherapy (yes/no), adjuvant hormonal therapy (yes/no), adjuvant radiation therapy (yes/no), surgical approach (partial/total mastectomy), sentinel lymph-node biopsy, and axillary lymph-node dissection. Education and dominant side (right/left) were obtained from the questionnaires.

### Statistical analysis

Single and multiple linear regression analyses were performed to investigate the association between preoperative grip strength and postoperative upper extremity impairments. The multiple linear regression analysis was adjusted for age, BMI, postoperative period, surgical approach, neoadjuvant chemotherapy, adjuvant chemotherapy, adjuvant radiation therapy, surgical approach, axillary lymph-node dissection, and matching of the dominant hand to the affected side. All statistical analyses were performed using R version 4.1.1. *p* values < 0.05 were considered statistically significant.

## Results

**Fig. 1:**
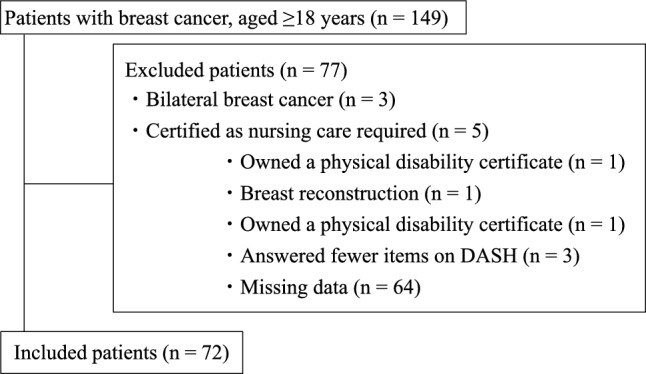
Flowchart of the selection process

Of the 149 recruited patients, 77 were excluded, and 72 were enrolled in this study (Fig. 1). Patient characteristics are presented in Table 1.

**Table 1:** Patient characteristics

	*n* = 72
Age (years)	62.0 ± 13.2
Female	72 (100.0)
BMI (kg/m^2^)	22.7 ± 3.4
Comorbidities	
High blood pressure	19 (26.4)
Diabetes mellitus	5 (6.9)
Musculoskeletal disease	7 (9.7)
Dominant side affected	38 (52.8)
Clinical stage	
0	7 (9.7)
I	32 (44.4)
II	24 (33.3)
III	7 (9.7)
IV	2 (2.8)
Postoperative period (months)	10.0 ± 3.6
Neoadjuvant chemotherapy, yes	12 (16.7)
Neoadjuvant hormonal therapy, yes	5 (6.9)
Adjuvant chemotherapy, yes	19 (26.4)
Adjuvant hormonal therapy, yes	44 (61.1)
Adjuvant radiation therapy, yes	23 (31.9)
Surgical approach	
Partial mastectomy	22 (30.6)
Total mastectomy	50 (69.4)
Sentinel node biopsy	53 (73.6)
Axillary lymph-node dissection	19 (26.4)
Preoperative grip strength of affected side (kg)	22.3 ± 5.2
Postoperative DASH score	12.1 ± 4.7

Data are presented as means ± SD or number of patients (percentages)

*SD* standard deviation, *BMI* body mass index, *DASH* disabilities of arm, shoulder and hand

Single linear regression analysis demonstrated that preoperative grip strength was significantly associated with postoperative DASH scores (*β* = − 0.74, 95% confidence interval [CI] − 1.39 to − 0.08, *p* = 0.03) (Table 2). In multiple linear regression analysis, preoperative grip strength was significantly associated with postoperative DASH scores after adjusting for covariates (*β* = − 1.27, 95% CI − 2.08 to − 0.48, *p* = 0.002) (Table 3). Higher preoperative grip strength was associated with better preoperative shoulder range of motion on the affected side in flexion and abduction (data not shown). No significant differences in preoperative patient characteristics were observed between the included and excluded patients (Online Appendix 1).

**Table 2:** Single linear regression analysis of the association between preoperative grip strength and postoperative DASH score

Variable	*β*	95% CI	*p* value
Preoperative grip strength of the affected side (kg)	− 0.74	− 1.39, − 0.08	0.03

*CI* confidence interval, *DASH* disabilities of arm, shoulder and hand

**Table 3:** Multiple linear regression analysis of the association between preoperative grip strength and postoperative DASH score

Variable	*β*	95% CI	*p* value
Preoperative grip strength of the affected side (kg)	− 1.27	− 2.08 to − 0.48	0.002
Age (years)	− 0.10	− 0.52 to 0.15	0.276
BMI (kg/m^2^)	− 0.34	− 1.38 to 0.69	0.511
Postoperative period (months)	− 0.54	− 1.62 to 0.54	0.319
Surgical approach (ref; partial mastectomy)			
Total mastectomy	− 1.63	− 9.81 to 13.08	0.776
Neoadjuvant chemotherapy, yes	− 4.61	− 15.50 to 6.28	0.400
Adjuvant chemotherapy, yes	− 3.85	− 13.65 to 5.95	0.435
Adjuvant radiation therapy, yes	0.88	− 9.98 to 11.73	0.872
Axillary lymph-node dissection	9.15	− 0.38 to 18.68	0.059
Dominant side affected	5.28	− 2.17 to 12.74	0.162

*CI* confidence interval, *BMI* body mass index, *DASH* disabilities of arm, shoulder and hand

## Discussion

To the best of our knowledge, this is the first study to investigate the association between preoperative grip strength and postoperative upper extremity impairments in patients with breast cancer. The results showed that preoperative grip strength was an independent predictor of postoperative upper extremity impairments after adjusting for demographic characteristics and treatment-related factors. The DASH scale has been used in some studies of patients with breast cancer with postoperative upper extremity impairments, and it is associated with health-related QOL [30]. Improvements in patient’s ability and symptoms assessed using the DASH scale, possibly leading to an improvement in postoperative QOL.

Several studies have reported that BMI, surgical approach, and adjuvant treatments are risk factors for upper extremity impairments in breast cancer survivors; however, limited studies have focused on the variable factors. Among these risk factors, BMI is a potential preoperative variable, whereas grip strength was the most significant factor in the present study. In addition, most previous studies included postoperative survivors [7, 13]. Smoot et al. reported grip strength as a risk factor for upper extremity impairments; however, grip strength and its relationship with upper extremity impairments were only evaluated postoperatively [20]. A strength of the present study is that it revealed an association between upper extremity impairments and preoperative factors. As grip strength is an indicator of overall muscle strength, it can be improved through prehabilitation [31].

This study showed that patients with higher preoperative grip strength had fewer postoperative upper extremity impairments. Grip strength can be used to indicate global muscle strength [23–25]. Moreover, preoperative grip strength can be a predictor of grip strength at 1 month postoperatively in patients with breast cancer [32]. Therefore, high preoperative grip strength may indicate high postoperative muscle strength. Patients with breast cancer experience postoperative upper extremity impairments, such as restricted range of motion and decreased upper extremity strength, which can interfere with activities of daily living (ADL) such as self-care and housework. Thus, maintaining high muscle strength reduces the load on the affected upper limb in ADL and prevents the onset of upper extremity impairment.

Our results suggest that the risk of upper extremity impairments can be predicted using the simple index of grip strength. Thus, improving preoperative grip strength through prehabilitation can help prevent postoperative upper extremity impairments. In addition, grip strength may be positively correlated with general activity level. Previous studies have shown an association between high activity levels and better shoulder range of motion in patients with breast cancer [33]. Hence, preoperative approaches for improving grip strength may positively affect the range of motion by improving activity levels. In the present study, more than 20% of patients reported moderate or greater difficulty in strength-dependent movements, such as carrying heavy objects. This may partly explain the effect of grip strength on the DASH scores. Upper extremity impairment is an important factor that worsens QOL in breast cancer survivors. Therefore, preoperative screening and individualized follow-up according to the predicted risks may help prevent QOL decline related to upper extremity impairments. This study highlights the importance of preoperative grip strength, suggesting that targeted resistance training focused on muscle strengthening could be an effective preoperative intervention. However, based on our results, it is not conclusive whether the interventions should target the upper extremity or global muscle strength. Previously, it was believed that upper extremity exercise after breast cancer surgery may cause or worsen lymphedema. Conversely, some studies have reported that upper extremity training after breast cancer surgery is safe and does not affect the development or worsening of lymphedema [26, 34]. We believe that future intervention studies are needed to clarify which intervention is necessary to improve preoperative grip strength.

This study has several limitations. First, a potential for selection bias exists, as this study was conducted at a single institution, and many patients were excluded because of missing data. The comparison of preoperative characteristics between excluded and included patients revealed no significant difference (Appendix 1). However, further studies involving multiple institutions are warranted. Second, the postoperative period of the study participants was not uniform. Although this was addressed in the analysis by adjusting for the postoperative period as a confounding variable, a uniform postoperative period should be considered in future studies. Third, we did not account for psychological or social variables. Patients with cancer often experience psychological and social problems after diagnosis [35, 36], which may affect postoperative upper extremity impairments. We plan to address these variables in future research. Fourth, we did not assess the preoperative upper extremity impairment status (preoperative DASH score). Patients with orthopedic problems on the affected side and those certified as requiring preoperative nursing care were excluded to remove cases with upper extremity impairments that could be caused by factors besides surgery. However, we did not assess the status of preoperative upper extremity impairment in all patients. Some patients may have had undetected preexisting impairments. Future studies should evaluate preoperative functional impairments in detail using DASH and investigate longitudinal changes before and after surgery. This evaluation would allow the application the results of the present study to patients with breast cancer undergoing surgical treatment.

In conclusion, this study revealed that preoperative grip strength may predict upper extremity impairments in patients with breast cancer. It is crucial to provide prehabilitation to maintain and improve muscle strength immediately after diagnosis. Additionally, an individualized follow-up protocol based on preoperative screenings to prevent postoperative upper extremity impairments is necessary.

## Supplementary Information

Below is the link to the electronic supplementary material.

Supplementary file1 (DOCX 20 kb)
