# Supplementary material for: Association between preoperative grip strength and postoperative upper extremity impairments in patients with breast cancer: a retrospective cohort study
Source: Breast Cancer. 2025 Apr 10;32(4):750–6. doi: 10.1007/s12282-025-01699-2 (PMC12174271; doi:10.1007/s12282-025-01699-2)
Supplement: Supplementary file 1 — Supplementary file1 (DOCX 20 kb) [file 12282_2025_1699_MOESM1_ESM.docx]

| **Appendix 1. Characteristics of the included and excluded patients** | | | |
| --- | --- | --- | --- |
| Variable | Included patients (n=72) | Excluded patients (n=77) | *p*-value |
| Age (years) | 62.04 ± 13.20 | 62.17 ± 14.26 | 0.96 |
| BMI (kg/m^2^) | 22.71 ± 3.41 | 22.64 ± 4.26 | 0.91 |
| Comorbidities |  |  | 0.26 |
| High blood pressure | 19 (26.4) | 20 (26.0) | 1.00 |
| Diabetes mellitus | 5 (6.9) | 6 (7.8) | 1.00 |
| Musculoskeletal disease | 7 (9.7) | 5 (6.5) | 0.67 |
| Dominant side affected | 38 (52.8) | 29 (51.8) | 1.00 |
| Clinical stage |  |  | 0.26 |
| 0 | 7 (9.7) | 14 (19.4) |  |
| 1 | 32 (44.4) | 33 (45.8) |  |
| 2 | 24 (33.3) | 21 (29.2) |  |
| 3 | 7 (9.7) | 2 (2.8) |  |
| 4 | 2 (2.8) | 2(2.8) |  |
| Neoadjuvant chemotherapy, yes | 12 (16.7) | 7 (9.1) | 0.25 |
| Neoadjuvant hormonal therapy, yes | 5 (6.9) | 1 (1.3) | 0.18 |
| Preoperative grip strength of  the affected side (kg) | 22.28 ± 5.16 | 20.23 ± 5.49 | 0.14 |
| data missing |  | 59 |  |

Data are expressed as mean ± SD or number of patients (percentages)

BMI, body mass index; DASH, Disabilities of Arm, Shoulder and Hand; SD, standard deviation
